# Tibiofemoral osteoarthritis affects quality of life and function in elderly Koreans, with women more adversely affected than men

**DOI:** 10.1186/1471-2474-11-129

**Published:** 2010-06-22

**Authors:** Inje Kim, Hyun Ah Kim, Young-Il Seo, Yeong Wook Song, David J Hunter, Jin Young Jeong, Dong Hyun Kim

**Affiliations:** 1 Department of Internal Medicine, Hallym University Sacred Heart Hospital, Anyang, Korea; 2 Department of Internal Medicine, Seoul National University Hospital, Seoul, Korea; 3 Division of Research, New England Baptist Hospital, Boston, USA; 4 Hallym Research Institute of Clinical Epidemiology, Chunchun, Korea; 5 Department of Social and Preventive Medicine, Hallym University, Chunchun, Korea

## Abstract

**Background:**

The prevalence of knee osteoarthritis(OA) in East Asia is as common for men and even higher for women than that reported in the Caucasian population. Since both population aging and economic growth have taken place at a much faster pace in Asian countries, such as South Korea, one would expect knee OA to become a major public health problem. However, few studies have examined the influence of knee OA on the quality of life (QoL) and physical function in Asia. The aim of this cross-sectional study is to investigate the influence of knee osteoarthritis (OA) on the quality of life (QoL), function and lower extremity physical performance and the gender difference in its influence in elderly community residents in Korea.

**Methods:**

Participants were from the population-based Hallym Aging Study (HAS). The mean age of the 504 study subjects was 70.2 years and 274 (54%) were women. Demographic information was obtained by questionnaire, and radiographic evaluations consisted of weight-bearing semi-flexed knee radiographs. Self-reported QoL and function were assessed using Western Ontario and McMaster Universities Osteoarthritis (WOMAC) Index and Short Form 12-item (SF-12). Performance-based lower extremity function was assessed using the tests consisting of standing balance, usual walk and chair stands. The odds ratios(ORs) for belonging to the worst quartile of WOMAC and physical performance test were calculated by logistic regression analysis in radiographic knee OA compared to non-OA after adjustment of confounders. Scores for SF-12 items were analyzed using general linear models and means adjusted for age, BMI and OA severity were compared.

**Results:**

Subjects with radiographic knee OA had significantly increased OR for belonging to the worst WOMAC quartile(for pain, 2.13,95% confidence interval[CI], 1.33-3.40, for stiffness, 2.94,95% CI,1.78-4.86, and for function, 2.97, 95% CI,1.83-4.81) and significantly worse SF-12 scores compared to non-OA after adjustment of age, BMI and sex. Women had worse WOMAC and SF-12 scores compared to men, regardless of the presence of radiographic knee OA after adjustment of age, BMI and OA severity. OA subjects had significantly worse performance score for usual walk and chair stands compared to non-OA subjects, but the ORs were no more significant after adjustment of sex.

**Conclusions:**

Knee OA negatively affects the QoL and physical function in both genders, but women are more adversely affected than men.

## Background

Knee osteoarthritis (OA) has a formidable societal and public health impact[1]. The prevalence of knee OA in East Asia is as common for men and even higher for women than that reported in the Caucasian population[2]. Since both population aging and economic growth have taken place at a much faster pace in Asian countries, such as South Korea, one would expect knee OA to become a major public health problem. However, few studies have examined the influence of knee OA on the quality of life (QoL) and physical function in Asia.

The prevalence of knee OA is higher in women compared to men and this gender difference seems more exaggerated among Asian women. A discrepancy in the total knee replacement (TKR) surgery rate between Korean men and women has been reported, with the age-standardized rate ratios in women compared to men ranging from 7.4 to 8.0[3]. In our previous study, the prevalence of radiographic knee OA was about 3.5 times higher, while that of symptomatic knee OA was 5.2 times higher in women[4]. The aim of this study was to examine the influence of radiographic knee OA, specifically tibiofemoral OA, on the QoL and function among elderly community residents in Korea. At the same time, the gender difference in the influence of knee OA on the QoL and lower extremity function was examined.

## Methods

### Subject

The Hallym Aging Study (HAS) is a prospective cohort study investigating the health of elderly community residents in Chunchun, a city about 120 km east of Seoul, Korea. This ongoing study commenced in 2004 with follow-up examinations scheduled every 3 years. Its methods have been described elsewhere in detail[4]. Briefly, eligibility criteria included an age of more than 50 years. Using Korean National Census data for 2000, 200 of 1,408 census tracts were sampled according to residential area[5]. Study subjects were selected so that those over 65 years old represented about 70% of the study cohort.

Nine hundred and eighteen subjects were surveyed in the first triennial examination. This cross-sectional study involved 702 subjects from the second triennial examination (64 subjects died, 49 moved, and 103 declined to participate or could not be contacted since the first examination). Of the 702 participants, 129 subjects declined knee radiographs and 69 subjects were excluded due to poor knee radiograph quality(n = 4), total knee replacement surgery(n = 3) or missing films due to clerical error(n = 62). The remaining 504 subjects were analyzed for this study. The ethics committee of Hallym University approved the study protocol. Written informed consent was obtained from all the study participants.

Demographic information was collected using a standard questionnaire. Knee pain was assessed by asking, "Have you had pain, aching, or stiffness lasting at least a month in your knee? All subjects also completed the WOMAC[6] and the SF-12 questionnaire for evaluating the pain, self-reported functional status and QoL.

### Physical performance test

Physical performance of lower extremity was tested using the Health ABC battery, as reported previously, with modifications[7]. Briefly, standing balance, a 6-m usual walk, and five repeated chair stands were tested. To test standing balance, the subjects were asked to attempt to keep their feet in side-by-side, semi-tandem, and tandem positions for 10 seconds each. The subjects were given a score of 0 if they could hold a side-by-side standing position for 10 seconds but were unable to hold a semi-tandem position for 10 seconds, a score of 1 if they could hold a semi-tandem position for 10 seconds but were unable to hold a full tandem position for more than 2 seconds, a score of 2 if they could stand in the full tandem position for 3 to 9 seconds, and a score of 3 if they could stand in the full tandem position for 10 seconds. For the 6-m usual walk test, a 6-m walk at the subject's normal pace was timed twice, and the time of the faster of the two walks was used for scoring. For repeated chair stands, the subjects were asked to fold their arms across their chests and to stand up and sit down five times as quickly as possible, and the length of time required was measured. For repeated chair stands and 6-m usual walks, we created quartiles based on the performance of the study subjects, with quartile 0 representing the worst and quartile 3 representing the best performance.

### Radiographic assessment

Radiographic evaluations consisted of weight-bearing anteroposterior, 14 × 17-inch, semi-flexed knee radiographs. A Plexiglas frame (SYNARC, San Francisco, CA, USA) was used to standardize knee positions, according to the manufacturer's recommendations. Each knee was graded using the Kellgren-Lawrence (K-L) grade[8]. Radiographic knee OA was defined as being present if the subject had a radiographic grade in the tibiofemoral joint of ≥K-L grade 2. All radiographs were read twice by one reader, an academically based rheumatologist (HAK). The reproducibility of intra-reader assessments was high (for OA versus no OA, = 0.89). Films allocated different K-L grades at the two readings were adjudicated by consensus between the original reader and a second reader(DJH).

### Statistical analysis

Because a sizable majority of subjects marked 0 for WOMAC subscales, they were categorized into quartiles 0-3 with quartile 3 representing the worst scores. To compare the pain, stiffness and function as measured with WOMAC between subjects with and without radiographic OA( K-L grade 0 or 1), we performed logistic regression analysis to estimate odd ratios and the associated 95% confidence interval (CI) for belonging to the worst quartile after adjustment of age, BMI and sex. Male and female OA subjects were compared after adjustment of age, BMI and OA severity. In addition, sex-specific quartiles were created and analyzed.

Mean scores for SF-12 items were analyzed using general linear models (GLM) to control for factors influencing SF-12 scores. The mean scores were compared between subjects with OA and subjects without OA entering age, BMI and sex as covariates. In addition, sex-specific comparisons were made between subjects with and without OA entering age and BMI as covariates. Comparison between male and female OA subjects were made entering age, BMI and OA severity defined as K-L grade as covariates.

The proportion of subjects belonging to each category of physical performance test was compared between subjects with and without radiographic OA using Chi-square test. The risks for belonging to the worst scores (0 and 1 were combined due to small subject size) and the worst quartile were compared for standing balance test and for 6-m usual walk and chair stands tests, respectively, using logistic regression analysis in OA subjects. Data were analyzed using SPSS software (version 15.0; SPSS Inc., Chicago, IL). *P *< 0.05 (two-tailed) was considered statistically significant.

## Results

The mean age of the 504 study subjects was 70.2 years and 274 (54%) were women. Those who declined to take radiographs were significantly older than the subjects who participated, but the percentage of women was not significantly different (mean age 73.4 years, 57% women). Radiographic and symptomatic knee OA(the presence of radiographic OA and knee pain) was present in 188(37.3%, 36[15.7%] in men, 152[55.5%] in women) and 122(24.2%, 17[7.4%] in men, 105[38.3%] in women) subjects, respectively(4).

OA subjects had significantly increased risk of belonging to the worst pain, stiffness and function quartile as measured with WOMAC compared to non-OA subjects after adjustment of age, BMI and sex (Table 1). In sex-specific analysis, while male OA subjects had only worse pain subscale score compared to male non-OA subjects, female OA subjects had worse scores for all 3 subscales compared to female non-OA subjects. It is of note that women had significantly higher risk for belonging to the worst quartile for all WOMAC subscales compared to men regardless of the presence of knee OA after adjustment of age, BMI and OA severity.

**Table 1:** Adjusted odds ratios for association between radiographic OA and worst WOMAC subscale quartiles

	Pain		Stiffness		function	
	
	Unadjusted OR (95% CI)	Adjusted OR (95% CI)	Unadjusted OR (95% CI)	Adjusted OR (95% CI)	Unadjusted OR (95% CI)	Adjusted OR (95% CI)
OA (vs non- OA)	3.71 (2.45-5.63)	2.13 (1.33-3.40)^†^	3.32 (2.20-5.01)	2.94 (1.78-4.86)^†^	5.07 (3.29-7.80)	2.97 (1.83-4.81)^†^
Men with OA (vs Men with non-OA) *	2.35 (1.11-4.94)	2.21 (1.03-4.74)^‡^	1.32 (0.59-2.96)	1.31 (0.57-3.01)^‡^	1.59 (0.73-3.43)	1.44 (0.65-3.19)^‡^
Women with OA (vs women with non-OA) *	2.58 (1.42-4.68)	2.15 (1.15-4.01)^‡^	2.57 (1.43-4.63)	2.45 (1.32-4.53)^‡^	3.00 (1.64-5.49)	2.56 (1.36-4.80)^‡^
Women with non-OA (vs men with non-OA)	2.48 (1.48-4.15)	2.64 (1.54-4.53)^‡^	3.28 (1.94-5.55)	3.42 (1.98-5.91)^‡^	2.48 (1.48-4.15)	2.95 (1.70-5.14)^‡^
Women with OA (vs men with OA)	4.35 (1.26-4.93)	5.19 (1.43-8.88)§	3.47 (1.16-10.39)	3.99 (1.25-12.77)^§^	3.16 (1.05-9.47)	4.11 (1.25-13.54)^§^

OA: subjects with radiographic tibiofemoral OA; non-OA: subjects with normal radiograph;

* Data were analyzed using sex-specific quartiles. † adjusted for age, BMI and sex, ‡ adjusted for age and BMI, § adjusted for age, BMI and OA severity.

The scores for the SF-12 items are shown in Table 2. Subjects with radiographic knee OA had significantly worse physical functioning, general health perception and physical component summary (PCS) scores compared to subjects without OA after adjustment of age, BMI and sex. Women had worse scores than men, regardless of the presence of OA. Bodily pain, mental health, emotional role and mental component summary (MCS) scores were significantly worse in female compared to male OA subjects after adjustment of age, BMI and OA severity.

**Table 2:** SF-12 scores in study subjects

	All		Men			Women		
	
	Non-OA	OA	Non-OA	OA		Non-OA	OA	
	**Adjusted**^1^	**Adjusted**^1^	**Adjusted**^2^	**Adjusted**^2^	**Adjusted**^3^	**Adjusted**^2^	**Adjusted**^2^	**Adjusted**^3^
Physical functioning	61.6	50.6 **	70.6	51.6 **	48.8	55.0 ‡	44.5 *	41.2
Physical role	70.0	65.5	78.7	69.2	66.4	63.8 ‡	58.8	56.6
Bodily pain	75.6	71.2	81.9	77.4	75.0	71.8 ‡	64.7	63.1 †
General health perception	46.3	38.7 **	48.9	40.7	37.8	44.6	36.6 *	37.0
Mental health	71.1	74.6	77.4	84.8	85.2	66.7 ‡	67.9	66.6 ‡
Emotional role	82.9	86.0	91.2	90.8	91.3	76.3 ‡	79.9	78.4 †
Vitality	34.1	31.8	41.4	32.7	33.2	29.1 ‡	26.6	25.0
Social functioning	87.3	85.1	91.1	87.2	87.1	84.7 †	82.0	81.0
Physical component score	63.4	56.5 **	70.0	59.7 *	57.0	58.8 ‡	51.1 *	49.5
Mental component score	68.9	69.3	75.3	73.9	74.2	64.2 ‡	64.1	62.8 ‡

OA:subjects with radiographic tibiofemoral OA; non-OA: subjects with normal radiograph.

Values are mean. Adjusted^1 ^: adjusted for age, BMI, sex, Adjusted^2 ^: adjusted for age, BMI, Adjusted^3 ^: adjusted for age, BMI ,OA severity;

* P < 0.05, ** P < 0.01 non-OA vs. ROA; † P < 0.05, ‡ P < 0.01 men vs. women

We performed Chi-square test to compare the percentage of subjects belonging to each physical performance category according to the presence of radiographic knee OA(Table 3). Female OA subjects had significantly worse functional categories compared to female non-OA subjects in all 3 tests performed, while in men there was no significant difference between OA and non-OA subjects. Because there may be inherent difference in lower extremity performance between genders, we did not compare the performance between men and women. Next, logistic regression analysis was used to estimate odd ratios of OA subjects for belonging to the worst functional categories after adjustment of confounders(Table 4). OA subjects had significantly worse performance score for usual walk and chair stands compared to non-OA subjects after adjustment of age and BMI. However, the ORs were no more significant after adjustment of sex. In sex-specific analysis, while male OA subjects had worse usual walk score compared to male non-OA subjects, there was no significant difference in ORs between female OA and non-OA subjects.

**Table 3:** Health ABC physical performance test categories in study subjects

		Men			Women		
	
	Performance score	Non-OA (N = 194)	OA (N = 36)	*p *Value	Non-OA (N = 122)	OA (N = 152)	*p *Value
Standing balance	0	11 (5.7)	3 (8.3)	0.093	6 (4.9)	11 (7.2)	0.001
	1	25 (12.9)	5 (13.9)		15 (12.3)	30 (19.7)	
	2	97 (50.0)	24 (66.7)		54 (44.3)	85 (55.9)	
	3	61 (31.4)	4 (11.1)		47 (38.5)	26 (17.1)	
Usual walk	0	43 (22.2)	14 (38.9)	0.163	22 (18.0)	45 (29.6)	0.007
	1	49 (25.3)	9 (25.0)		26 (21.3)	43 (28.3)	
	2	51 (26.3)	7 (19.4)		33 (27.0)	37 (24.3)	
	3	51 (26.3)	6 (16.7)		41 (33.6)	27 (17.8)	
Chair stands	0	42 (21.6)	15 (41.7)	0.079	23 (18.9)	46 (30.3)	0.013
	1	49 (25.3)	8 (22.2)		25 (20.5)	43 (28.3)	
	2	52 (26.8)	7 (19.4)		35 (28.7)	34 (22.4)	
	3	51 (26.3)	6 (16.7)		39 (32.0)	29 (19.1)	

For standing balance, the subjects were given a score of 0-3 as described in the method. For the 6-m usual walk test and repeated chair stands, quartiles based on the performance of the study subjects were created.

Zero represents the worst and 3 represents the best performance. Values are the number (%). Distribution of categories between non-OA and OA was compared with Chi-square test.

**Table 4:** Adjusted odds ratios for association between knee OA and worst lower extremity physical performance categories

	Standing balance		Usual walk		Chair stands	
	
	Unadjusted OR (95% CI)	Adjusted OR (95% CI)	Unadjusted OR (95% CI)	Adjusted OR (95% CI)	Unadjusted OR (95% CI)	Adjusted OR (95% CI)
OA (vs non-OA)	1.48(0.95-2.33)	1.30(0.81-2.08)^†^	2.70(1.78-4.09)	2.26(1.46-3.50)^†^	2.63(1.73-4.02)	2.30(1.47-3.58)^†^
		0.87(0.50-1.50)^‡^		1.26(0.77-2.06)^‡^		1.33(0.81-2.19)^‡^
Men with OA (vs men with non-OA)*	1.03(0.39-2.67)	0.96(0.36-2.56)^†^	2.68(1.26-5.68)	2.32(1.07-5.01)^†^	2.28(1.07-4.86)	1.94(0.87-4.32)^†^
Women with OA (vs women with non-OA)*	1.60(0.88-2.92)	1.04(0.54-2.01)^†^	1.66(0.94-2.93)	1.01(0.54-1.90)^†^	2.03(1.12-3.66)	1.26(0.66-2.42)^†^

* Data were analyzed using sex-specific quartiles. † adjusted for age, BMI, ‡ adjusted for age, BMI, sex.

## Discussion

In these Korean community residents aged 50 years and older, the presence of radiographic tibiofemoral OA significantly impaired the QoL and function. This study is in line with a report from Singapore, which revealed substantially worse bodily pain and physical function scores among patients with hip or knee OA compared to the general population[9]. Women were more adversely affected than men.

It is known that the prevalence of knee OA in East Asia among men is similar to that reported for Caucasian Americans, but that it is higher among Asian women[2,10]. For instance, in the Beijing OA study, the age-standardized prevalence of symptomatic knee OA in Chinese women was 2.9 times higher than that of men in Beijing, whereas the corresponding prevalence ratio was 2.0 in the Framingham study[2]. Many reports from Caucasian populations have also shown that female patients with OA had a higher level of pain compared to men[11]. In this study, we found that women had worse WOMAC and SF-12 scores than men, regardless of the presence of knee OA. Corroborating our data, a recent report involving 2,282 Japanese community residents demonstrated that female sex was a strong risk factor for knee pain even in the subgroup without radiographic knee OA[12]. In addition, in a community study of 819 subjects with knee pain, women had a lower occurrence of radiographic knee OA compared to men, which suggests that knee pain may arise at a location distinct from the knee joint preferentially in women[13]. Genetic polymorphisms such as the W200/G324 haplotype of *FRZB*, which is significantly associated with both hip and knee OA in Caucasian women but not in men may account for the gender differences in different ethnic groups[14]. Kneeling and squatting are strong risk factors for knee OA, and this life style factor might account for greater functional disability as well as the prevalence of knee OA in Koreans [15-17]. Korean women tend to squat more often than men in daily activities, such as for the toilet and house chores. Knee flexion for squatting and kneeling may be more important factor determining knee pain and knee function in Korea compared to Western countries in which ambulation is more important. Lateral and skyline radiographs were not taken in this study, thus, the prevalence of patellofemoral OA could not be evaluated. Patellofemoral OA is also more common among Asian females[17], thus it may be a factor explaining the gendere difference in pain and function. Other risk factors for knee OA, such as the obesity, lower educational level or manual occupation was also more frequent among females[4], thus these factors may account for the increase of knee pain, knee OA and poor function among women.

A test battery including a 6-min walk, a stair climb, a lifting and carrying task, and getting into and out of a car has been validated and used to evaluate physical activity in patients with knee OA[18,19]. In another study, factors associated with a poor chair-stand test in a predominantly female knee OA cohort over 3 years were studied, with age and proprioceptive inaccuracy increasing and strength, self-efficacy, and aerobic exercise decreasing the risk of a poor outcome[20]. In this study, we used the Health ABC battery, which included two items--chair stands and usual walk tests--applied in previous studies in addition to standing balance. We found that although female OA subjects did worse compared to female non-OA, the difference was lost after adjustment of age and BMI. In addition, the higher OR of OA subjects for belonging to the worst categories compared to non-OA subjects became non-significant after adjusting sex. This may be due to the fact that lower extremity function measured by this battery is more strongly influenced by age and sex than by the presence of OA.

Our study has strengths and limitations. This is the first population-based study on the influence of knee OA on physical function and QoL in Asian subjects with knee OA. Second, we used a physical performance test as well as questionnaires to evaluate the functional aspect of knee OA. All the questionnaires used have been validated in Korean. On the other hand, the limited number of study subjects might have prevented some of the measures from reaching significance (*e*.*g*., only 36 male subjects with radiographic knee OA). In addition, only A-P knee radiographs were included, so patellofemoral OA could not be evaluated.

## Conclusions

In these Korean community residents aged 50 years and older, the presence of radiographic tibiofemoral OA significantly impaired the QoL and function. This study demonstrates that in common with Caucasian patients, Korean patients with knee OA have substantial pain and worse physical performance, with women more adversely affected than men.

## Competing interests

The authors declare that they have no competing interests.

## Authors' contributions

All authors read and approved the final manuscript.

IK performed the statistical analysis and wrote the manuscript. HAK conceived of the study, read radiographs, supervised data analysis and wrote the manuscript. YIS conceived of the study and revised the manuscript critically. DJH adjudicated the radiograph, and supervised data analysis. WYS, JYJ and DHK performed the epidemiological survey and performed the statistical analysis.

## Pre-publication history

The pre-publication history for this paper can be accessed here:

http://www.biomedcentral.com/1471-2474/11/129/prepub
